# CT lymphangiography of the thoracic duct in mice: direct mesenteric *versus* popliteal lymph node puncture

**DOI:** 10.1186/s41747-025-00568-z

**Published:** 2025-02-18

**Authors:** Shimpei Kato, Haruto Sugawara, Toshihiro Furuta, Osamu Abe, Hiroyuki Akai

**Affiliations:** 1 Department of Radiology, Institute of Medical Science, The University of Tokyo, Tokyo, Japan; 2 Department of Radiology, Graduate School of Medicine, The University of Tokyo, Tokyo, Japan

**Keywords:** Lymph nodes, Lymphography, Mice (inbred BALB C), Thoracic duct, X-ray microtomography

## Abstract

**Background:**

To evaluate the efficacy of computed tomography (CT) lymphangiography after direct mesenteric lymph node injection for thoracic duct (TD) visualization in mice.

**Methods:**

Twelve female BALB/c mice were injected with 35 μL of iodinated contrast medium (iomeprol 350 mgI/mL) into the mesenteric (mesenteric group) or popliteal (popliteal group) lymph nodes. CT images were acquired before injection and 1 min, 3 min, 5 min, 10 min, and 15 min after injection using a micro-CT scanner. Contrast ratios (CRs) were measured at the cisterna chyli and three levels of the TD (diaphragm, carina, and venous angle). Two experienced radiologists qualitatively assessed images as good, fair, or poor.

**Results:**

The mesenteric group had significantly higher mean (± standard deviation) CRs than the popliteal group for all examined regions at 1 min after injection: cisterna chyli (14.01 ± 4.77 *versus* 1.47 ± 1.21, *p* < 0.001), diaphragm (7.28 ± 2.50 *versus* 0.85 ± 0.61, *p* = 0.0011), carina (10.33 ± 3.42 *versus* 0.44 ± 0.40, *p* < 0.001), and venous angle (6.26 ± 2.02 *versus* 0.79 ± 0.75, *p* < 0.001). For the TD between the cisterna chyli and the diaphragm, 6/6 mice in the mesenteric group showed strong enhancement, whereas 5/6 mice in the popliteal group showed minimal or no enhancement. The visual scores of the mesenteric group were significantly higher than those of the popliteal group for all the evaluated regions (*p* = 0.002).

**Conclusion:**

CT lymphangiography *via* mesenteric lymph node injection provides better imaging of the TD in mice than popliteal lymph node injection.

**Relevance statement:**

This study enhances TD visualization in mice, advancing preclinical research on lymphatic disorders and improving translational applications for better clinical diagnostics and treatments.

**Key Points:**

- Mesenteric lymph node injection improved the efficacy of TD CT lymphangiography in mice.
- Mesenteric injection provided significantly better TD visualization than popliteal injection.
- Enhanced TD visualization in mice advances preclinical research on lymphatic diseases.

**Graphical Abstract:**

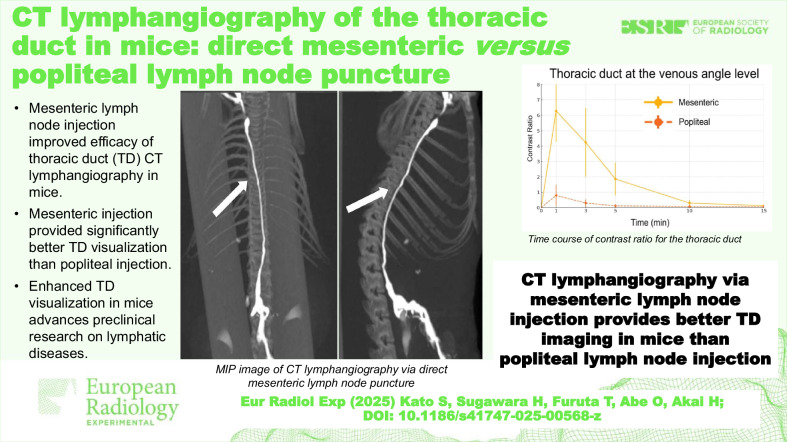

## Background

Chylothorax is the accumulation of lymphatic fluid in the pleural cavity. It causes respiratory distress and malnutrition and poses a significant clinical challenge [1]. Accurate visualization of the thoracic duct (TD) is crucial for diagnosing and treating chylothorax, enabling targeted therapeutic interventions such as TD embolization or ligation [2, 3]. TD imaging, especially lymphangiography, is essential for evaluating chylothorax pre- and postoperatively [4–6].

In human medicine, mesenteric lymphangiography allows visualization of the TD and its branches [7, 8]. The feasibility and efficacy of this technique have also been studied in veterinary medicine. In cats, successful visualization of the TD and its anatomical variations through computed tomography (CT) lymphangiography has been achieved by injecting iohexol into mesenteric lymph nodes under ultrasound guidance [9]. Similarly, in dogs with idiopathic chylothorax, preoperative and postoperative assessment of the TD and detection of abnormal TD drainage patterns using percutaneous CT lymphangiography of the mesenteric lymph nodes under ultrasound guidance have been reported [10]. However, data on its use in mice are lacking.

Mice are widely used in the study of human disease pathophysiology because of the ease of genetic manipulation and the availability of various disease models [11]. However, the anatomical features of the TD and the pathophysiology of chylothorax in mice are not well understood. There are anatomical and physiological similarities between the lymphatic circulations of mice and higher mammals. In addition, their rapid reproductive cycles and manipulable genomes make them cornerstones of biomedical research [11]. Therefore, establishing a TD imaging method in mice is important for basic research and developing therapeutic strategies.

In a previous study, although satisfactory visualization of the abdominal lymphatic structures—namely, lymph nodes, lymphatic vessels, and the cisterna chyli—was achieved, the degree of TD visualization was not assessed [12]. We hypothesized that the relatively long distance between the popliteal lymph nodes and the TD might negatively affect its visualization, and that mesenteric lymph node puncture could be advantageous in this regard. This study aimed to investigate the feasibility of CT lymphangiography for visualizing the TD in mice by direct injection of iodinated contrast medium into mesenteric lymph nodes.

## Methods

All animal experiments were conducted following institutional guidelines and approved by the institutional animal research committees (see “Declarations”).

### Animals

Twelve female BALB/c mice were purchased and maintained in a specific pathogen-free facility. All mice weighed approximately 20 g and had ad libitum access to food and water.

### CT lymphangiography

The mice were divided into two groups: a popliteal lymph node group (*n* = 6) and a mesenteric lymph node group (*n* = 6). For the popliteal group, twenty microliters of 25 mg/mL indocyanine green were injected subcutaneously into the left rear footpad, and baseline CT images of the same mice were acquired. All images were obtained using micro-CT for small animals (CosmoScan FX, Rigaku Corporation, Tokyo, Japan). The mice were anesthetized with 4% isoflurane in the air, and 1.5% isoflurane was used for imaging. They were placed in the prone position fixed in a mouse holder before the injection of the contrast medium. The scanning parameters were as follows: tube voltage, 90 kVp; tube current, 88 µA; exposure time, 18 s; field of view, 46 × 46 mm^2^; and pixel size, 0.09 × 0.09 mm^2^.

The skin of the popliteal fossa was incised to visualize the popliteal lymph nodes, which were punctured directly and slowly injected with 35 µL of the iodine contrast material (iomeprol; iodine concentration, 350 mgI/mL; Iomeron 350; Bracco Medical Imaging, Milan, Italy). In our previous study, the peak contrast ratio (CR) of lumbar-aortic lymph nodes following popliteal lymph node administration was observed at 2 min [12]. Considering the rapid metabolism in mice, it is possible that this peak may be observed in less than 2 min. Therefore, we performed CT imaging under the same conditions at 1 min, 3 min, 5 min, 10 min, and 15 min after the injection.

In the mesenteric group, under anesthesia, the abdomen was opened, and baseline CT images of the same mice were acquired. The mesenteric lymph nodes near the cecum were punctured under direct visualization, followed by the injection of 35 μL of contrast medium. The mice remained with their abdomens open and were positioned on the CT table immediately before injection. Imaging was commenced at 1 min after the injection, with subsequent imaging at 3 min, 5 min, 10 min, and 15 min.

### Image analysis

The time courses for obtaining the CT lymphangiographic images were quantitatively analyzed. Circular regions of interest, as large as possible, were placed at the cisterna chyli and the diaphragm, carina, and venous angle levels of the TDs in the baseline images, and the average density within the regions of interest were measured using the ImageJ software by the radiologist with 10 years of experience. The same process was performed for images obtained at 1 min, 3 min, 5 min, 10 min, and 15 min after the contrast injection. The measured density of the lymph nodes in the baseline image was designated as S_pre_, and the density of the lymph nodes after contrast injection was designated as S_post_. The CR was calculated as follows:

$${{\rm{CR}}}=({{{\rm{S}}}}_{{{\rm{post}}}}\,-\,{{{\rm{S}}}}_{{{\rm{pre}}}})/{{{\rm{S}}}}_{{{\rm{pre}}}}$$

**Fig. 1:**
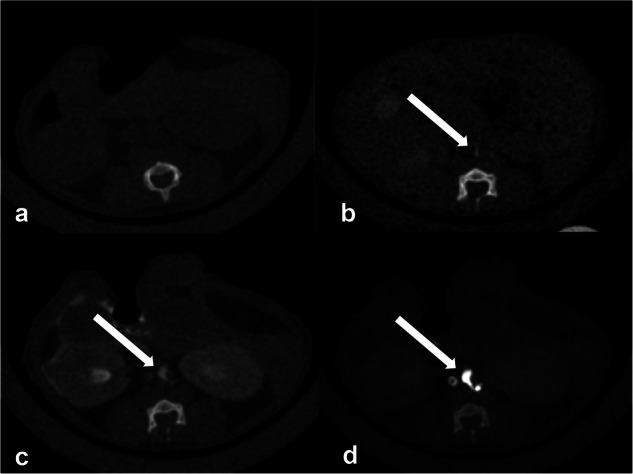
**a** Pre-contrast image. **b** Representative image with a poor score (minimal or no enhancement). **c** Representative image with a fair score (visible, but not strong enhancement). **d** Representative image with a good score (strong enhancement). Arrows in the figure show the cisterna chyli

**Fig. 2:**
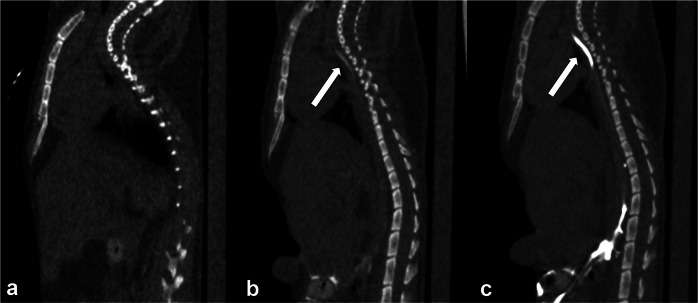
Sagittal images of the TD. **a** Representative image with a poor score (minimal or no enhancement). **b** Representative image with a fair score (visible, but not strong enhancement). **c** Representative image with a good score (strong enhancement)

For the qualitative analysis, two radiologists (with 21 years and 19 years of experience, respectively) evaluated the visibility of the cisterna chyli and the TD on axial images at three segments at 1 min and 10 min after contrast injection. The segments of the TD assessed were between the cisterna chyli and the diaphragm, the diaphragm, and the carina, and the carina and the venous angle. The visual scores based on a three-point scale were as follows: good: strong enhancement; fair: visible, but not strong enhancement; and poor: minimal or no enhancement. The scoring scale used in this study was consistent with that used in the previous research [12]. Examples of each category are provided in Figs. 1 and 2.

### Statistical analysis

The CRs of the cisterna chyli and the three segments of the TD for the popliteal and mesenteric injection groups were compared at 1 min and 10 min after injection using a *t*-test. For the qualitative study, the visual scores of the TD of the popliteal and mesenteric injection groups were compared using the Wilcoxon rank sum test. The statistical analyses were conducted using R software (ver. 4.2.2; R Foundation for Statistical Computing, Vienna, Austria), and statistical significance was set at *p* < 0.05.

## Results

**Fig. 3:**
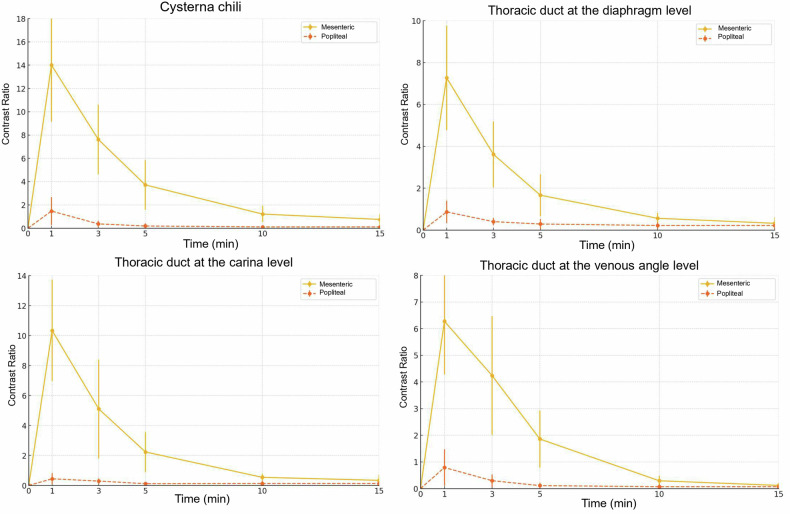
Time course of CR for the cisterna chyli and the TD. The mesenteric lymph node puncture group showed the highest CR at 1 min after injection, which decreased over time. The mesenteric lymph node puncture group had higher CRs than the popliteal lymph node puncture group at all time points. Bars represent mean ± standard deviation. CR, Contrast ratio

Figure 3 shows the time course of the CRs after direct injection into the popliteal and mesenteric lymph nodes. For the mesenteric lymph node group, the CR for all the regions peaked in the CT images at 1 min after injection. For the popliteal lymph node group, the CRs were low at all time points. The mesenteric lymph node group showed a significantly higher CR of the cisterna chyli at 1 min after injection (14.01 ± 4.77 [mean ± standard deviation]) than the popliteal lymph node group (1.47 ± 1.21, *p* < 0.001). Similarly, the mesenteric lymph node group had significantly higher CRs than the popliteal lymph node group at the diaphragm (7.28 ± 2.50 *versus* 0.85 ± 0.61, *p* = 0.0011), carina (10.33 ± 3.42 *versus* 0.44 ± 0.40, *p* < 0.001), and venous angle levels (6.26 ± 2.02 *versus* 0.79 ± 0.75, *p* < 0.001) of the TD at 1 min after contrast administration.

Table 1 presents the qualitative analysis results. The visual scores were significantly higher for the mesenteric than for the popliteal lymph node group at 1 min after injection for the cisterna chyli and the three segments of the TD between the cisterna chyli and the diaphragm, the diaphragm, and the carina, and the carina and the venous angle (*p* = 0.002, 0.002, 0.002, and 0.002, respectively). For the TD between the cisterna chyli and the diaphragm level, 6 of 6 mice (100%) in the mesenteric injection group showed strong enhancement, and no mice had poor visibility (minimal or no enhancement). In contrast, 5 of 6 mice (83%) in the popliteal injection group showed minimal or no enhancement of the same segment.

**Table 1:** Visual score distribution in the qualitative assessment of cisterna chyli and TD at 1 min and 10 min after injection

	Popliteal direct puncture	Mesenteric direct puncture	*p*-value
1 min after injection			
Cisterna chyli	0/3/3	6/0/0	0.0024^a^
Cisterna chyli—diaphragm	0/1/5	6/0/0	0.0018^a^
Diaphragm—carina	0/3/3	6/0/0	0.0024^a^
Carina—venous angle	0/3/3	6/0/0	0.0024^a^
10 min after injection			
Cisterna chyli	0/0/6	0/5/1	0.0067^a^
Cisterna chyli—diaphragm	0/0/6	0/3/3	0.071
Diaphragm—carina	0/0/6	0/3/3	0.071
Carina—venous angle	0/0/6	0/5/1	0.0067^a^

For each cell of the table, the number of mice scored as being good, fair, or poor is listed

^a^ Statistically significant

As shown in Fig. 3, the CRs peaked at 1 min and subsequently decreased over time in both groups, reaching substantially lower values at 10 min. At 10 min after injection, the visual scores were significantly higher for the mesenteric than for the popliteal injection group only for the cisterna chyli and for the TD segment between the carina level and the venous angle level (*p* = 0.007 for both). There were no significant differences in the TD between the cisterna chyli and the diaphragm and between the diaphragm and the carina (*p* = 0.071 and 0.071, respectively). While all regions in all mice were rated as “good” at 1 min, none of the regions received a “good” rating at 10 min. Instead, they were evaluated as “fair” or “poor.”

**Fig. 4:**
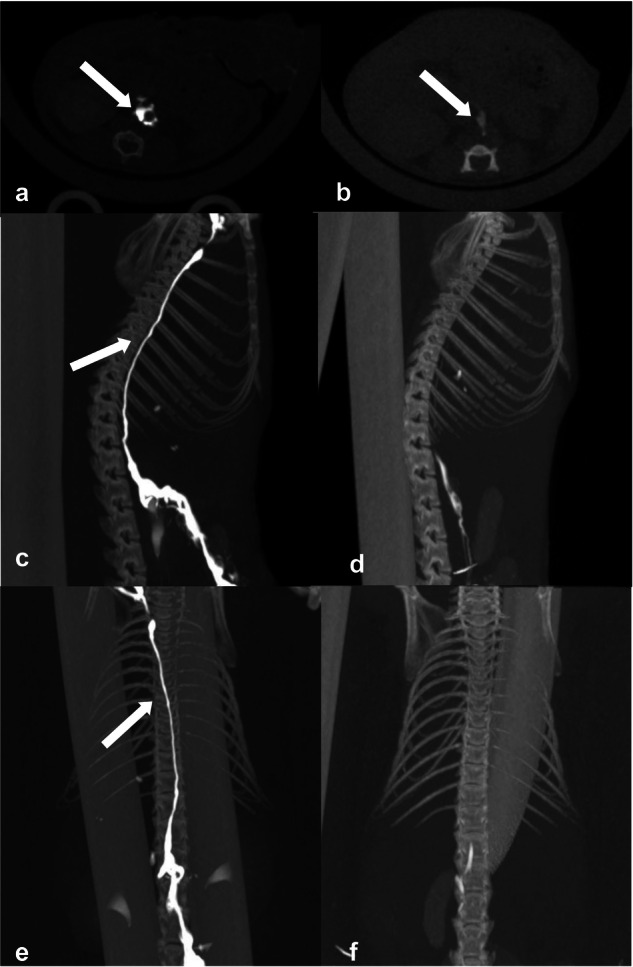
CT lymphangiography in the mice after (**a**) direct mesenteric and (**b**) popliteal lymph node injections. The cisterna chyli was more strongly enhanced after the direct mesenteric lymph node puncture. **c**, **e** MIP image of CT lymphangiography after direct mesenteric lymph node injection. **d**, **f** MIP image of CT lymphangiography after direct popliteal lymph node injection. CT lymphangiography after the direct mesenteric lymph node injection depicted the lymphatic pathway more clearly. All images were obtained 1 min after injection. CT, Computed tomography; MIP, Maximum intensity projection

Figure 4 shows the differences in the visibility of the lymphatic system of the TD of the mesenteric and popliteal lymph node groups. As suggested by the quantitative and qualitative results, the contrast effect of cisterna chyli was stronger with the mesenteric puncture than with the popliteal injection on axial CT images (Fig. 4). This trend was further emphasized when maximum intensity projection images were acquired. The cisterna chyli and TD segments of the popliteal lymph node group were barely visible, whereas the structures and their interrelationships and the entire lymphatic system of the mesenteric lymph node group were clearly visible (Fig. 4).

## Discussion

This study demonstrated that CT lymphangiography is more feasible and effective for visualizing the TD in mice after mesenteric than after popliteal lymph node injection of iodinated contrast medium. Our findings are consistent with those reported in larger animals, such as cats and dogs [9, 10], indicating that mesenteric lymph node injection provides clear and detailed imaging of the TD.

Both quantitatively and qualitatively, the mesenteric lymph node group demonstrated superior outcomes compared to the popliteal lymph node group, exhibiting significantly higher CR of TD at 1 min after injection, as well as improved visualization of TD segments and the cisterna chyli. This suggests that the mesenteric approach enables more efficient distribution of the contrast medium throughout the lymphatic system, enhancing opacification of the TD. Given that both groups demonstrated peak values at 1 min, dilution effects would explain the higher CR observed in the mesenteric approach compared to the popliteal approach. As described in a previous study [13], the lymphatic system operates as a one-way network of vessels and nodes where fluid mixing occurs. The popliteal route encounters multiple mixing points through nodes and naturally experiences greater dilution as the contrast medium mixes with a larger volume of lymphatic fluid along its extended path. In contrast, the mesenteric injection travels a shorter route with fewer mixing points, thereby preserving higher contrast concentration and explaining the higher CR while maintaining similar enhancement timing.

Our findings have important implications for preclinical applications. In preclinical studies, accurate and detailed imaging of the TD in mice is essential to evaluate the efficacy of novel therapeutic interventions for lymphatic disorders such as chylothorax. In addition, a recent study reported that TD abnormalities may be a cause of retroperitoneal edema and abdominal pain, emphasizing the growing importance of TD imaging in clinical settings [14]. The molecular mechanisms involved in the pathogenesis of chylothorax can be elucidated using genetically modified mice [15]. Furthermore, mouse TD imaging can be used to evaluate the effects of lymphangiogenic agents or physical stimuli that improve lymphatic flow [15, 16]. The ability to reliably visualize the TD using CT lymphangiography in mice increases the applicability of these studies and may enable a more effective translation of discoveries to human medicine.

Traditionally, lymphangiography in human clinical practice has mainly been performed via ultrasound-guided inguinal lymph node puncture, while mesenteric lymph node puncture was documented in only a very limited number of case reports [7, 8]. To establish a standardized method of mesenteric lymphangiography, this preclinical study using a larger sample size and statistical analysis could be beneficial. We believe it would be noteworthy that this study has elucidated the time-intensity curve for CT lymphangiography via mesenteric lymph node puncture, whereas previous literature [9, 10], in which CT lymphangiography via mesenteric lymph node puncture was performed on cats and dogs, only documented a single time-point CT scan after mesenteric lymph node injection.

Lipiodol has predominantly been used as the contrast agent for lymphangiography in clinical practice, which potentially poses a risk of non-targeted embolization when it enters the systemic circulation [17, 18]. Due to this, CT lymphangiography with water-soluble iodine contrast has been reported very recently [19]. Unlike lipiodol, which is a highly viscous oil-based contrast agent, water-soluble iodinated contrast agents tend to have a much faster washout, making it crucial to understand the optimal imaging timing for improved visualization of the TD. We believe that, in this context, the time-intensity curve from this study provides valuable insights into determining the appropriate imaging timing for TD visualization in CT lymphangiography, serving as an important pre-clinical study.

A previous noninvasive quantification study of TD lymph flow in pigs [20] reported a peak enhancement approximately 180 s after contrast administration, which is significantly later than the 60-s peak observed in the present study in mice. This discrepancy is likely attributable to inter-species differences in lymphatic circulation speed. In general, animals tend to have faster metabolism as their body size decreases. Therefore, it is hypothesized that mice may have faster lymphatic flow compared to pigs. Additionally, the infusion protocol used by Molloi et al (0.1 mL/kg at 0.1 mL/s) may have contributed to a delayed peak compared with the single-bolus administration in this study. Although the difference of injection sites, namely inguinal lymph nodes *versus* popliteal/mesenteric lymph nodes, potentially would have affected transit times, given that the peak time was similar among popliteal injection and mesenteric injection in our study, this effect would be minimal.

In addition, this is the first study to explore mesenteric lymphangiography in mice. Mice are widely used in research due to their short breeding cycle, small size, low cost, and ease of genetic modification. Their genome has also been well studied, and their genetic similarity to humans makes them advantageous as models for specific diseases and therapies [21]. For example, the genetic signaling involved in lymphatic malformations has been thoroughly analyzed in recent years, and disease models using knockout mice have become accessible [22]. Therefore, we believe that by establishing a method for CT lymphangiography using mesenteric node puncture in mice, we can contribute to future research on lymphatic-related diseases.

In both mice and humans, magnetic resonance imaging (MRI) lymphangiography using gadolinium-based contrast agents is considered an alternative to CT lymphangiography for visualizing the lymphatic system [23–26]. MRI has several advantages, including superior soft-tissue contrast, the absence of ionizing radiation, and the capability for dynamic imaging. However, MRI lymphangiography in mice has some limitations, such as long durations of imaging, high costs, and a propensity for motion artifacts. Our previous results revealed that MRI lymphangiography in mice was inadequate, especially for depicting lymphatic vessels distant from the injection site [12].

This study has several limitations. First, the relatively small sample size may limit the generalizability of the results. Second, the injection technique requires precise anatomical knowledge and skills, which may limit its application by less experienced practitioners. Standardizing the procedure and providing detailed training could mitigate this issue. Third, mesenteric lymph node puncture requires an invasive procedure involving laparotomy. Laparoscopic lymph node procedures are less invasive than laparotomy and serve as effective diagnostic and therapeutic approaches. In veterinary medicine, laparoscopic mesenteric lymph node lymphangiography has been successfully performed in dogs [27], demonstrating reduced invasiveness. Although such lymphangiographic studies have not been conducted in humans, several clinical studies have documented successful laparoscopic lymph node biopsies, suggesting the feasibility of this approach [28, 29]. Furthermore, both CT-guided lymphangiography [8] and endoscopic ultrasound-guided fine needle aspiration/biopsy of intra-abdominal lymph nodes [30] have been reported in human patients. These established minimally invasive techniques suggest that mesenteric lymphangiography could potentially be performed with significantly reduced invasiveness compared to traditional laparotomy. Fourth, it is possible that the true peak time was not accurately captured because the earliest imaging time point was 1 min, a limitation imposed by procedural and technical constraints. In the future, a dynamic CT or MRI study with a more detailed temporal profile would be desirable, but for the primary objective—the comparative assessment of TD enhancement originating from popliteal *versus* mesenteric lymph nodes—the current temporal resolution would remain sufficient. Finally, this study focused only on healthy mice. Conditions such as inflammation or tumors may affect lymphatic flow and contrast agent distribution and impact the effectiveness of the imaging technique.

In conclusion, this study demonstrated that mesenteric lymph node injection provides better quantitative and qualitative imaging of the TD in CT lymphangiography than popliteal injection in mice. These findings support the utility of the mesenteric lymph node injection method and offer a promising approach for improving the diagnosis and treatment of lymphatic disorders. Future research should aim to further optimize this technique and explore its application across various preclinical models to enhance our understanding and management of lymphatic diseases.

## Data Availability

The datasets used and/or analyzed during the current study are available from the corresponding author upon reasonable request.

## Abbreviations

CR: Contrast ratio
CT: Computed tomography
MRI: Magnetic resonance imaging
TD: Thoracic duct
